# Effect of potato glycoside alkaloids on mitochondria energy metabolism of *Fusarium solani*, the root rot pathogen of *Lycium barbarum*

**DOI:** 10.3389/fmicb.2024.1512027

**Published:** 2025-01-29

**Authors:** Ruiyun Li, Bin Wang, Wei Chen, Chongqing Zhang, Nan Li, Yupeng Wang, Yuke Yan, Yuyan Sun, Jing He

**Affiliations:** ^1^College of Forestry, Gansu Agricultural University, Lanzhou, China; ^2^Wolfberry Harmless Cultivation Engineering Research Center of Gansu Province, Lanzhou, China

**Keywords:** *Fusarium solani*, TMT technology, potato glycoside alkaloids, energy metabolism, mitochondrion

## Abstract

*Fusarium solani* is a widely distributed pathogenic fungus that can cause soil borne diseases in various plants and is also one of the main pathogenic bacteria of *Lycium barbarum* root rot. This study employed tandem mass labeling (TMT) quantitative proteomics technology to investigate the antifungal mechanism of potato glycoside alkaloids (PGA) against *Fusarium solani*. We elucidated the antifungal mechanism of PGA from the perspective of mitochondrial proteome molecular biology. Furthermore, we identified and annotated the differentially expressed proteins (DEP) of *F. solani* under PGA stress. A total of 2,412 DEPs were identified, among which 1,083 were significantly up-regulated and 1,329 significantly down-regulated. Subsequent analysis focused on five DEPs related to energy metabolism for verification at both protein and gene levels. Gene Ontology (GO) function analysis revealed that the DEPs were primarily involved in the integral component of the membrane, intrinsic component of the membrane, pyridine-containing compound metabolic processes, carbon-oxygen lyase activity, and the endoplasmic reticulum, with a notable enrichment in membrane components. Furthermore, a total of 195 pathways were identified through KEGG analysis, with significant enrichment in critical pathways including pentose and glucuronate interconversions, propanoate metabolism, various types of N-glycan biosynthesis, the pentose phosphate pathway, and carbon fixation in photosynthetic organisms. The results from both parallel reaction monitoring (PRM) and real-time RT-qPCR were consistent with the overall trends observed in TMT proteomics, thereby confirming the validity of the TMT proteomics analysis. These findings indicate that PGA inhibits the growth of *F. solani* by impacting mitochondrial energy metabolism. This study reveals the antifungal mechanism of PGA from the perspective of energy metabolism, providing a theoretical basis for the development and application of PGA as a biopesticide.

## 1 Introduction

*Lycium barbarum* is a perennial shrub that belongs to the Solanaceae family and the *Lycium* genus. It is a main economic tree species in Gansu Province. It is widely distributed in the northwest saline alkali desert areas (Zeng et al., 2023). The fruit of *L. barbarum* is sweet and juicy, commonly consumed raw, juiced, or brewed into tea in local traditions. It possesses high nutritional and medicinal value (Zeng et al., 2023; Li et al., 2018; Liu et al., 2022). However, root rot is one of the primary diseases that hinders the development of the wolfberry exhibition industry, leading to a decline in the quality of wolfberry (Bai et al., 2020). *L. barbarum* root rot is a prevalent soil-borne disease that primarily affects the roots and stems of the plant, impeding the transport and absorption of water and nutrients. This results in poor plant growth, reduced quality and yield of *L. barbarum* fruit, and in severe cases, can lead to plant wilting and death. It is classified as a typical vascular disease (He et al., 2023). *Fusarium* is identified as the main pathogen responsible for root rot (El-Saadony et al., 2021), with *Fusarium solani* being the most widely distributed species. Currently, the prevention and control of *L. barbarum* root rot rely primarily on chemical methods. Chemical fungicides not only disrupt the ecological balance of soil, but also lead to excessive pesticide residues in *L. barbarum* berries and induce resistance of pathogenic fungi. Therefore, developing green, safe, and efficient biological antifungal agents to combat *L. barbarum* root rot is of significant importance.

Potato glycoside alkaloids (PGA) are a class of steroidal alkaloids characterized by the presence of 1–4 monosaccharides. These compounds are secondary metabolites predominantly found in the green parts of potato tubers, stems, and leaves (Song et al., 2023). The concentration of glycoside alkaloids in potato tubers decreases progressively as the potatoes mature (Koffi et al., 2017). PGA has garnered significant attention due to its abundant availability, cost-effectiveness, environmental safety, and broad-spectrum antifungal properties. Previous research has identified α-solanine and α-theophylline as the primary antifungal constituents of PGA. Notably, α-solanine exhibits a potent antifungal effect against Botrytis cinerea, with its inhibitory intensity being dose-dependent (Sun et al., 2014). Previous studies have utilized the method of inhibiting the growth of flat mycelium to evaluate the antifungal activity of PGA against *Cercosporella brassicae*, *Alternaria porri*, *A. solani*, and *F. solani*. The results indicated that PGA exhibited inhibitory effects on the growth of all four pathogens. Notably, the inhibitory effect on *C. brassicae* and *A. porri* was particularly pronounced, while the effects on *A. solani* and *F. solani* were comparatively weaker (Zhao et al., 2013). The antifungal efficacy of PGA against *F. solani*, *Marssonina juglandis*, and *Capnodium leaophilum* was assessed using the growth rate method, revealing that PGA possessed notable antifungal activity against these three fungi, with the strongest inhibition observed against *F. solani*, which also enhanced cell membrane permeability (Duo et al., 2017). Additionally, it has been reported that PGA can inhibit both mycelial growth and spore development of *Curvularia trifolii* (Xu et al., 2023), diminish the virulence of *Pectobacterium brasiliense* (Joshi et al., 2021), and inhibit reactive oxygen species (ROS) metabolism in *F. sulphureum*, thereby exerting antifungal effects (Li et al., 2022). Our previous study demonstrated that PGA inhibited respiration and ROS metabolism in *F. solani*, disrupted mitochondrial structure, and ultimately suppressed pathogen growth (Ding et al., 2023). Furthermore, PGA can attenuate tricarboxylic acid cycle flux by modulating the expression of key genes, contributing to its antifungal effects (Zhang et al., 2024).

Previous studies on the antifungal effects of PGA have primarily concentrated on physiological and biochemical aspects, with a notable lack of research involving proteomics. However, recent advancements in proteomics research methods have emerged rapidly. Tandem Mass Tag (TMT)-based quantitative proteomics technology is one of the most powerful analytical techniques available, offering the highest throughput, minimal systematic error, and robust capabilities for the quantitative analysis of differentially expressed proteins (DEPs). This technology is characterized by high sensitivity, excellent separation capacity, and substantial throughput. Furthermore, TMT technology has been employed to systematically analyze the expression changes of mitochondrial proteins from a proteomic perspective, enabling the identification of differential proteins and the verification of DEPs expression levels. This approach aims to elucidate the antifungal mechanism of PGA on *F. solani* from a molecular biology standpoint.

## 2 Materials and methods

### 2.1 Test materials

#### 2.1.1 Tested pathogens

*F. solani* was isolated from diseased *L. barbarum* plants, which were identified and preserved in the Forest Protection Science Laboratory at Gansu Agricultural University. Prior to use, the isolates were activated on Potato Dextrose Agar (PDA) medium (Potato 200 g, glucose 20 g, agar 20 g and water 1,000 mL) at 25°C and subsequently stored at 0 to 4°C for later application.

#### 2.1.2 Preparation of PGA

The extraction of PGA follows the method described by Huang et al. (2012). First, weigh 100 g of potato sprout powder and add 400 mL of 5% acetic acid (chemically pure). Stir the mixture using a magnetic stirrer (EMS-13, Tianjin Ounuo Instrument Co., Ltd.) for 1 h. Subsequently, employ a vacuum filtration pump [SHZ-D (III), North South Science Instrument (Beijing) Technology Co., Ltd.] to filter the mixture, and extract the residue twice with 200 mL of 5% acetic acid. Combine the filtrate from these three extractions. Adjust the pH to 10.0 using ammonia water, and then perform an extraction with water-saturated n-butanol (200 mL) in three portions. Collect and combine the extracts, and evaporate them using a rotary evaporator (RV10V, Suzhou Sainz Instrument Co., Ltd.) at 65°C and 40 rpm for 1 h. Finally, dissolve the residue in 20 mL of methanol (chemically pure) to obtain the PGA extraction mother liquor with a concentration of 88.10 mg/mL.

According to the principle of color development, PGA (solanine and chalcone) can form a purple-red complex with formaldehyde in an acidic environment. The intensity of the color is positively correlated with the PGA content. The absorbance of the solution at 530 nm is measured, and the PGA concentration of the sample solution is determined using a standard curve based on previous research results from the group. The regression equation for the standard curve is *y* = 0.3628x+0.0256, with an *R*^2^ value of 0.9583. For PGA measurement, 1 mL of the PGA extract is taken, and the method for constructing the standard curve is followed. Methanol (instead of the sample solution) is adjusted to zero, and the absorbance (A) at 530 nm is measured to calculate the PGA content using the formula: (FW)


(1) P⁢G⁢A⁢(m⁢g/g)=(V×C/W)×100

where V is the volume of the sampled liquid (mL), C is the concentration of the sample solution as determined by the standard curve (mg/mL), and W is the fresh weight of the sample (g).

The repeatability test was conducted on the PGA extract, revealing an average PGA content of 0.503 mg/g and a relative standard deviation (RSD) of 3.25% (< 10%). This indicates good repeatability of the method, based on five replicates, and confirms its suitability for the quantitative analysis of PGA.

#### 2.1.3 Determination of EC_50_ value

The EC_50_ was determined using the mycelium growth rate method. Based on the results of the preliminary concentration screening experiment, plates containing different concentrations of PGA (1.0, 2.0, 4.0, 8.0, and 16.0 mg/mL) were prepared. *F. solani* was inoculated with fungal cake using a sterile puncher (φ = 6 mm), with sterile water serving as the control (CK). The cultures were maintained at 25°C and the experiment was repeated three times. From this, the toxicity regression equation and the half maximal effect concentration (EC_50_).


(2) Inhibitedgrowthrate(%)=

$$\frac{\left(c\,o\,n\,t\,r\,o\,l\,c\,o\,l\,o\,n\,y\,d\,i\,a\,m\,e\,t\,e\,r-t\,r\,e\,a\,t\,m\,e\,n\,t\,c\,o\,l\,o\,n\,y\,d\,i\,a\,m\,e\,t\,e\,r\right)}{\left(c\,o\,n\,t\,r\,o\,l\,c\,o\,l\,o\,n\,y\,d\,i\,a\,m\,e\,t\,e\,r-b\,a\,c\,t\,e\,r\,i\,a\,l\,c\,a\,k\,e\,d\,i\,a\,m\,e\,t\,e\,r\right)}\times 100\%$$

Convert the inhibition rate into a probability value and the mass concentration into the logarithm of the mass concentration. Designate the logarithm of the mass concentration of each agent as x and the probability value of the inhibition rate as y. Subsequently, perform data processing to derive the toxicity regression equation *y* = 0.9385x–0.0511, and the correlation coefficient was *R*^2^ = 0.9407. Finally, determine the effective medium mass concentration EC_50_ value of the test agent.

#### 2.1.4 Observation of mycelium by scanning electron microscope

Samples for scanning electron microscopy were prepared following the method outlined by Vujanovic et al. (2019). A total of 0.1 g of mycelium from both the control group and the PGA treatment group were fixed in a 2.5% glutaraldehyde phosphate buffer (pH 7.2) for 16 h. The samples were then rinsed three times with phosphate buffer, with each rinse lasting 15 min. Gradient dehydration was conducted sequentially using 30, 50, 70, 90, 95, and 100% ethanol. Subsequently, the ethanol in the samples was replaced with isoamyl acetate. The samples were then dried at the critical point, vacuum coated, and finally examined and imaged using a scanning electron microscope (JSM-5600LV, Japan Electron Optics Co., Ltd.).

#### 2.1.5 Preparation of mitochondria

Mitochondria were prepared using the method described by Zhang et al. (2023) and Zhan et al. (2021), employing differential centrifugation. *F. solani* was inoculated on PDA and cultured at 25°C for 7 d to allow for the maturation of *F. solani* spores. A spore suspension of *F. solani* (1 × 10^6^ spores/mL) was prepared, and 50 μL of this suspension was added to 30 mL of Potato Dextrose Broth (PDB) medium (Potato 200 g, glucose 20 g and water 1,000 mL) for shaking culture at 25°C and 160 rpm. After 2 d of culture, the mycelium was transferred to PDB medium with a final concentration of 3.88 mg/mL. The mycelium was collected after an additional 2 d of continuous culture at 25°C and 160 rpm. The mycelium was then filtered through sterile gauze, washed three times with sterile water, and dried using a vacuum pump. The mycelium was ground to crush the cells; following this, centrifugation was performed at 4°C at 1,500 × *g* for 5 min, after which the supernatant was collected and the precipitate discarded. The supernatant was subsequently centrifuged at 12,000 × *g* at 4°C for 20 min. The mitochondrial precipitate was then resuspended in mitochondrial extraction buffer.

#### 2.1.6 TMT analysis of mitochondrial protein of *F. solani*

The TMT analysis of mitochondrial proteins from *F. solani* was conducted by Shanghai Personal Biotechnology Co., Ltd. Mitochondrial samples of *F. solani* were extracted using the SDT [4%(w/v) SDS, 100 mM Tris/HCl pH7.6, 0.1M DTT] lysis method, followed by protein quantification using the BCA method. Each sample underwent trypsinization via the Filter Aided Proteome Preparation (FASP) method (Wiśniewski et al., 2009). The resulting peptides were desalted with a C18 cartridge, then lyophilized and reconstituted in 40 μL of a 0.1% formic acid solution. For peptide quantification, 100 μg of peptides were taken from each sample and labeled according to the instructions provided in the Thermo Scientific TMT labeling kit. The samples were then separated using an Easy nLC HPLC system with a nanoliter flow rate. Chromatographic separation was followed by analysis using a Q-Exactive mass spectrometer. The raw data for mass spectrometry analysis are RAW files, and the software Mascot 2.2 and Proteome Discoverer 1.4 are used for inventory identification and quantitative analysis. The mass spectrometry proteomics data have been deposited to the ProteomeXchange Consortium^1^ via the iProX partner repository with the dataset identifier PXD058927.

#### 2.1.7 Bioinformatics analysis methods

In this experiment, differentially expressed proteins (DEPs) were screened, and a volcano plot was generated based on the criteria of a fold change (FC) greater than 1.2 for up-regulation or less than 0.83 for down-regulation, with a *p*-value of less than 0.05 (determined by T-test or other methods). The annotation of the target protein set was conducted using Blast2GO, which can be summarized in four main steps: sequence alignment (Blast), Gene Ontology (GO) item extraction (Mapping), GO annotation (Annotation), and supplementary annotation through InterProScan (Annotation Augmentation). Additionally, KEGG pathway annotation analysis of the target protein set was performed using KAAS (KEGG Automatic Annotation Server) software. Fisher’s Exact Test was employed to compare the distribution of each GO category (or KEGG pathway, or Domain) within the target protein set against the overall protein set. Furthermore, enrichment analysis of GO annotations and KEGG pathway or Domain annotations was conducted on the target protein set.

#### 2.1.8 Verification of DEPs

##### 2.1.8.1 Parallel reaction monitoring (PRM) verification

A sample of approximately 100 μg of protein was prepared by adding DTT to achieve a final concentration of 100 mM. The mixture was boiled in a water bath for 15 min and subsequently cooled to room temperature. Next, 200 μL of UA buffer (8 M Urea, 150 mM Tris-HCl, pH 8.0) was added, and the solution was mixed before being transferred to a 10 kDa ultrafiltration tube. Centrifugation was performed at 4°C and 14,000 × *g* for 30 min. An additional 200 μL of UA buffer was added, and centrifugation was repeated under the same conditions, after which the filtrate was discarded. Subsequently, 100 μL of iodoacetamide [IAA, (50 mM IAA in UA)] was added, shaken at 600 rpm for 1 min, and protected from light at room temperature for 30 min, followed by centrifugation at 4°C and 14,000 × *g* for 20 min. This washing step was repeated three times with the addition of 100 μL of UA buffer and centrifugation at 4°C and 14,000 × *g* for 20 min. Following this, 100 μL of NH_4_HCO_3_ buffer (50 mM) was added, and centrifugation was conducted at 4°C and 14,000 × *g* for 20 min, repeated twice. Next, 40 μL of NH_4_HCO_3_ buffer (containing Trypsin at an enzyme-to-substrate ratio of 1:50) was added, shaken at 600 rpm for 1 min, and incubated at 37°C for 16 h. After incubation, a new collector tube was used, and the sample was centrifuged at 4°C and 14,000 × *g* for 15 min. The filtrate was collected after a subsequent centrifugation at 4°C and 14,000 × *g* for 30 min, with an additional 40 μL of NH_4_HCO_3_ buffer (50 mM) added. The peptides resulting from enzymatic hydrolysis were desalted and freeze-dried, then redissolved in 0.1% FA, with the peptide concentration determined by OD_280_. The peptide information suitable for PRM analysis was imported into the software Xcalibur for PRM method setup. Approximately 1 μg of peptides from each sample was mixed with 20 fmol of standard peptide (PRTC: ELGQSGVDTYLQTK) for detection. The samples were separated using high-performance liquid chromatography and analyzed by PRM mass spectrometry with a Q-Exactive HF mass spectrometer (Thermo Scientific). PRM-related proteins are shown in Table 1.

**TABLE 1:** PRM quantitative protein.

Number	Accession	Description	Gene name
1	XP_046138580.1	Phosphoglucomutase	FP2
2	KAJ4234247.1	Glucokinase	GLK1
3	KAJ4221670.1	Fructose-bisphosphate aldolase 1	FBA1
4	KAJ4221476.1	Phosphoglycerate kinase	PGK1
5	KAJ4235649.1	Cytochrome c oxidase subunit 6B	COX12

##### 2.1.8.2 Real-time fluorescent quantitative PCR

RT-qPCR technology was employed for further functional verification analysis at the transcriptional level. Total RNA was extracted from the control group and the treatment group using RNAsimple Total RNA Kit [DP419, Tiangen Biochemical Technology (Beijing) Co., Ltd], and all RNA samples were reverse transcribed into cDNA using the Hifair AdvanceFast One-step RT-gDNA Digestion SuperMix for qPCR. Primers for each candidate gene were designed using NCBI online software. The mycelium cDNA served as the template in conjunction with the Hieff UNICON Advanced qPCR SYBY Master Mix reagent, with Actin utilized as the internal reference gene. Quantitative analysis was conducted using a fluorescence quantitative PCR instrument, following a reaction procedure of 95°C for 30 s, 95°C for 10 s, and 60°C for 30 s, repeated for 40 cycles. The specificity of the amplification products was assessed based on the dissolution curve, with three replicates for each sample. The relative expression of the target gene was calculated utilizing the 2^–ΔΔCt^ method. RT-qPCR protein primer sequences are shown in Table 2.

**TABLE 2:** RT-qPCR primer sequence.

Number	Gene name	Description	Forward primer (3′–5′)	Reverse primer (5′–3′)
1	PGK1	Phosphoglycerate kinase	AAGGACAAGGAGGGCAACAAGAC	GTTCCAAAGGCGTCGTTGATGTAG
2	FBA1	Fructose-bisphosphate aldolase 1	GTTTGCCACACAGATGCCTACAC	TCACCGTTGCTCACACCCTTG
3	FP2	Phosphoglucomutase	TCTCCTCTGGTAGCCGAATTGTTG	GGCGAACTTGACCTCCTCCTTC
4	FDH1_2	Formate dehydrogenase (NAD+)	GTTTGCCACACAGATGCCTACAC	GACGCCCTCACCAACAGACTC
5	LPD1	Dihydrolipoamide dehydrogenase precursor	AGAAGCAGGGTATGGAGTTCAAGC	GCGACGAGGACGACATCAGAC

### 2.2 Data analysis

All data were analyzed by one-way ANOVA using SPSS 22.0, and the data were plotted by Origin 2021.

## 3 Results

### 3.1 Determination of EC_50_

**FIGURE 1:**
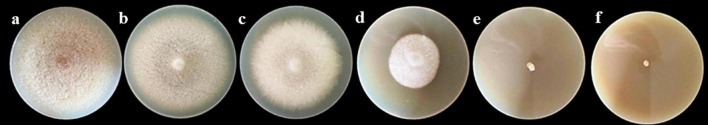
Illustrates the effects of various concentrations of PGA on the growth of *F. solani* colonies. The concentrations are represented as follows: **(A)** Control (CK), **(B)** 1.0 mg/mL, **(C)** 2.0 mg/mL, **(D)** 4.0 mg/mL, **(E)** 8.0 mg/mL, and **(F)** 16.0 mg/mL.

Different concentrations of PGA extracts (1.0, 2.0, 4.0, 8.0, and 16.0 mg/mL) exhibited varying inhibitory effects on *F. solani*. As the concentration of PGA treatment increased, the colony diameter progressively decreased (Figure 1), with significant differences observed compared to the control group (*P* < 0.05). The inhibition rate of PGA against *F. solani* increased in a dose-dependent manner, reaching a maximum of 100% at a concentration of 16.0 mg/mL. The virulence regression equation was expressed as *y* = 0.9385x–0.0511, with a correlation coefficient of *R*^2^ = 0.9407, indicating a strong linear relationship among the variables in the regression equation and confirming the feasibility of the test. When the value of y is 50%, the half-effect concentration (EC_50_) was determined to be 3.87 mg/mL (Table 3).

**TABLE 3:** Determination of antifungal activity of different concentrations of PGA against *F. solani*.

Treatment	Concentration (mg/mL)	Concentration logarithm (x)	Colony diameter (cm)	Inhibitory rates (%)	Probability value (y)
CK	/	/	7.41	/	/
PGA	1.0	0	7.33	1.09	−0.0511
	2.0	0.30103	6.63	10.45	0.231417
	4.0	0.60206	3.68	50.30	0.513933
	8.0	0.90309	0.36	95.14	0.79645
	16.0	1.20412	0	100	1.078967

### 3.2 Effects of PGA on the surface morphology of pathogen hyphae

**FIGURE 2:**
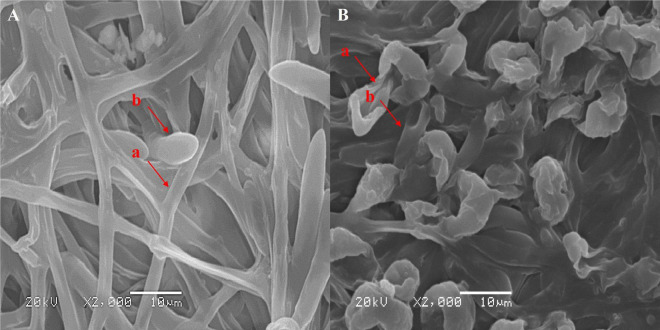
Scanning electron micrograph of mycelium morphology. **(A)** Sterile water treatment control, **(B)** PG A treatment. **(a)** hyphae, **(b)** spore.

The SEM results indicated that the mycelium of the control group exhibited a uniform thickness and lacked any noticeable defects, while the spore surface morphology appeared normal (Figure 2A). In contrast, following PGA treatment, the mycelium of *F. solani* displayed signs of swelling and curling, with the spore surface showing pronounced depressions (Figure 2B). These observations suggest that PGA inhibits the normal energy metabolism of *F. solani*. The energy supply within the hyphae and spore cells is inadequate to fulfill the typical growth and differentiation requirements of these cells. Consequently, their adaptability to environmental changes diminishes, making it challenging for them to adjust their morphology in a timely manner. This results in the development of abnormal hyphae and spore morphology.

### 3.3 Protein quantification and analysis

**FIGURE 3:**
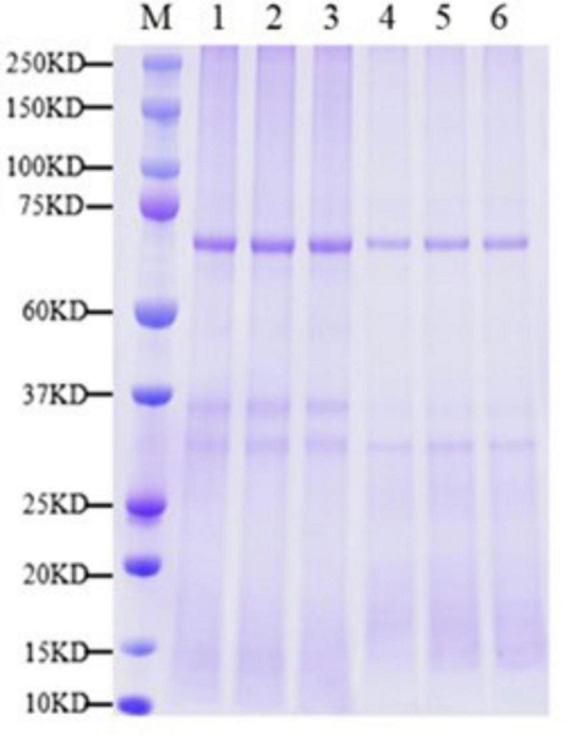
Sample SDS-PAGE. M represents marker; 1–3 were PGA treatment; 4–6 for CK. Each treatment had three biological replicates.

**FIGURE 4:**
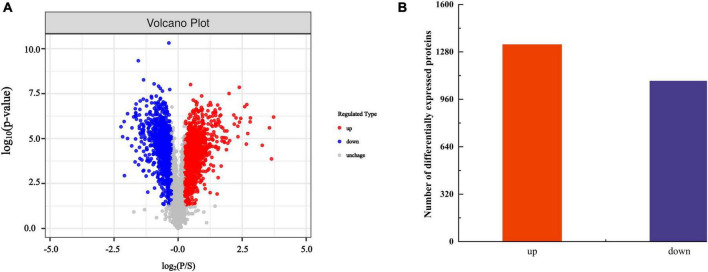
Statistics of DEPs. **(A)** The volcano map comparing PGA and CK shows red dots representing up-regulated DEPs and blue dots indicating down-regulated DEPs, while gray dots depict proteins with no differential expression changes. **(B)** Summary statistics of differentially expressed proteins.

By analyzing the expression changes of DEP after PGA treatment, we can deeply understand the functions and interactions of these proteins in *F. Solani*, and provide new perspectives and ideas for their bacteriostatic mechanism. The protein molecular weight distribution in both the PGA treatment group and the control group was comparable, exhibiting clear and evenly distributed protein bands with no significant diffusion observed (Figure 3). These results indicated that the samples from each group demonstrated good parallelism, maintained protein integrity, and showed no evident degradation. This suggests that the samples are suitable for future proteomic sequencing, protein identification and DEPs analysis. Additionally, the results revealed that, compared to the control group, 1,083 DEPs were up-regulated and 1,329 DEPs were down-regulated following PGA treatment (Figure 4). By analyzing the role of DEPs in metabolic pathway, we can reveal the abnormal changes of metabolic pathway after PGA treatment.

### 3.4 GO functional enrichment analysis of DEPs

**FIGURE 5:**
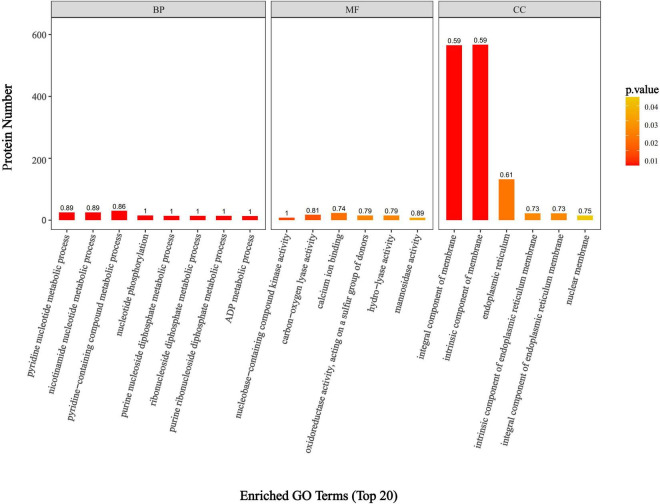
Enrichment statistics of DEPs in PGA vs. CK group.

GO annotation results indicated that the DEPs were predominantly enriched in biological processes (BP), molecular functions (MF), and cellular components (CC). The biological processes primarily involved pyridine nucleotide metabolism, nicotinamide nucleotide metabolism, and the metabolism of pyridine-containing compounds (Figure 5). These GO results suggest that DEPs are significantly enriched in various nucleotide metabolism biological processes, which may disrupt the energy metabolism rate of *F. solani*, affect its energy balance and stability, and hinder its ability to maintain normal cellular function.

### 3.5 KEGG enrichment analysis of DEPs

**FIGURE 6:**
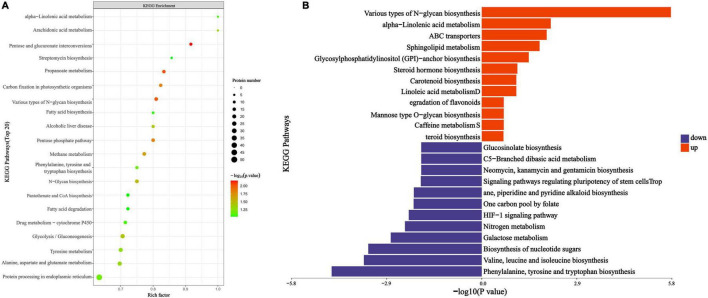
PGA vs. CK KEGG pathway enrichment map. **(A)** Bubble color indicates the significance of the enriched KEGG pathway. **(B)** Pathway categories enriched by ordinate KEGG, the statistical results of differential proteins under each KEGG pathway in abscissa; among them, red represents up-regulation and blue represents down-regulation.

To elucidate the overall characteristics of metabolic pathway enrichment among all DEPs, we evaluated the significance level of protein enrichment within the KEGG metabolic pathways. The analysis revealed a total of 195 pathways, among which several key pathways exhibited substantial differential protein enrichment, including the mutual transformation of pentose and glucuronic acid, propionic acid metabolism, various types of N-glycan biosynthesis, the pentose phosphate pathway, and carbon fixation in photosynthetic organisms (Figure 6A). Notably, the enrichment of differential proteins was most pronounced in three pathways: the mutual conversion of pentose and glucuronic acid, propionic acid metabolism, and various types of N-glycan biosynthesis, all of which play critical roles in energy metabolism.

Following KEGG metabolic pathway annotation, we analyzed the DEPs that were up-regulated and down-regulated, with the results illustrated in Figure 6B. The up-regulated DEPs were primarily enriched in pathways associated with the biosynthesis of phenylalanine, tyrosine, and tryptophan; the biosynthesis of valine, leucine, and isoleucine; nucleotide sugar biosynthesis; galactose metabolism; and nitrogen metabolism, among others. Conversely, the down-regulated DEPs were mainly enriched in various types of N-glycan biosynthesis and α-linolenic acid metabolism. Our analysis indicates that PGA treatment interferes with the energy metabolism pathways of *F. solani*, including lactose metabolism, nitrogen metabolism, and various types of N-glycan biosynthesis pathways.

### 3.6 Pathway analysis related to energy metabolism

**FIGURE 7:**
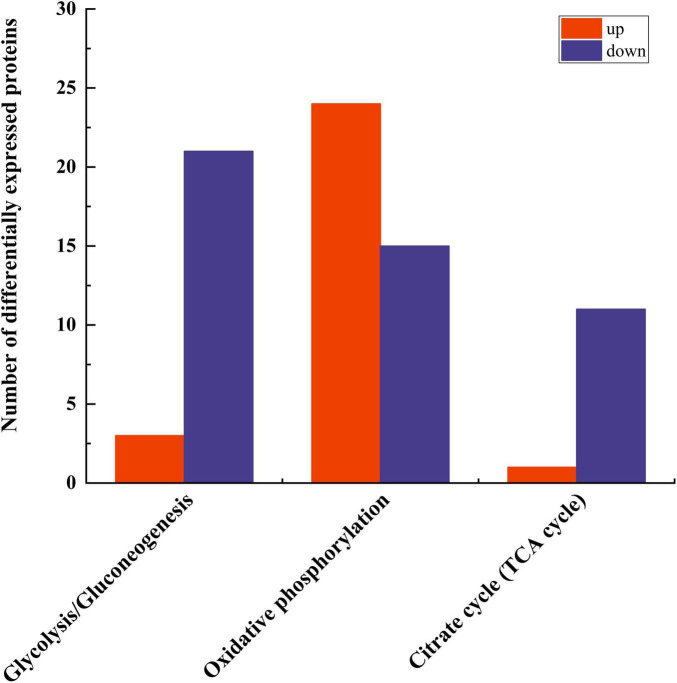
Differential protein statistics of pathways related to energy metabolism.

Three pathways related to energy metabolism were further examined, including the tricarboxylic acid cycle, glycolysis/gluconeogenesis, oxidative phosphorylation, and pyruvate metabolism, resulting in the identification of a total of 75 differential proteins (Figure 7). The number of down-regulated DEPs in these three pathways exceeded that of the up-regulated DEPs. The specific proteins are shown in Table 4.

**TABLE 4:** Pathway enrichment analysis of differential protein pathway.

Accession	Protein description	Regulated type	Folds	*p*-value
**Glycolysis/gluconeogenesis**				
KAJ3460530.1	Hypothetical protein MRS44_011397	Up	1.23759908	0.000889915
KAJ3469958.1	Hypothetical protein MRS44_000057	Up	1.05159239	0.155675838
KAJ4227054.1	Fructose-1,6-bisphosphatase	Up	1.01215369	0.713850924
KAJ3465376.1	Hypothetical protein MRS44_006034	Down	0.82509374	3.05887E–06
KAJ3467366.1	Hypothetical protein MRS44_004930	Down	0.80680684	0.000620567
KAJ4216690.1	Hypothetical protein NW759_009263	Down	0.7833298	3.20275E–05
KAJ3466824.1	Hypothetical protein MRS44_004388	Down	0.77210166	0.037350979
KAJ4205842.1	Dihydrolipoamide dehydrogenase precursor	Down	0.74787102	2.52016E–06
KAJ4220075.1	Hexokinase	Down	0.73307775	5.34577E–07
KAJ4222655.1	Formate dehydrogenase (NAD+)	Down	0.64291509	3.21864E–05
KAJ3462558.1	Hypothetical protein MRS44_007344	Down	0.61212393	9.08175E–06
KAJ4222180.1	Hypothetical protein NW759_006603	Down	0.60867332	7.47705E–06
KAJ4233040.1	GlucosE–6-phosphate isomerase	Down	0.59752279	7.14851E–07
KAJ4234247.1	Glucokinase	Down	0.59592741	1.75024E–07
KAJ3468704.1	Hypothetical protein MRS44_002769	Down	0.59064309	0.000831282
KAJ4205829.1	Hypothetical protein NW759_014467	Down	0.57478783	2.1E–05
KAJ4224181.1	Phosphopyruvate hydratase	Down	0.56454731	9.7898E–06
KAJ4235663.1	Hypothetical protein NW759_000749	Down	0.46406379	1.92474E–06
KAJ4235821.1	Hypothetical protein NW759_000904	Down	0.45270836	0.000194959
KAJ4229869.1	Triosephosphate isomerase	Down	0.43107143	0.000173363
KAJ4210794.1	Pyruvate kinase, variant 2	Down	0.42586017	3.93497E–05
XP_046138580.1	Phosphoglucomutase	Down	0.39442702	2.147E–06
KAJ4221670.1	Fructose-bisphosphate aldolase 1	Down	0.38395625	3.1938E–06
KAJ4221476.1	Phosphoglycerate kinase	Down	0.3211469	1.18841E–07
**Oxidative phosphorylation**				
YP_005088125.1	Cytochrome c oxidase subunit 3 (mitochondrion)	Up	3.05820084	1.20325E–06
KAJ3453979.1	Hypothetical protein MRS44_018611	Up	2.07024719	1.31735E–05
KAJ4228829.1	H^(+)^-transporting V0 sector ATPase subunit e	Up	1.94354037	0.000213983
KAJ4228970.1	Hypothetical protein NW759_003691	Up	1.87520625	1.82875E–07
KAJ3470770.1	Hypothetical protein MRS44_000869	Up	1.70182759	0.000996077
KAJ4208125.1	v-type proton ATPase 16 kDa proteolipid subunit 2	Up	1.67684135	0.00024295
KAJ3462825.1	Hypothetical protein MRS44_007611	Up	1.63733624	0.000777276
KAJ3471178.1	Hypothetical protein MRS44_001277	Up	1.60769733	5.79496E–05
YP_005088127.1	NADH: ubiquinone oxidoreductase subunit 6 (mitochondrion)	Up	1.59617177	0.004788767
YP_005088108.1	Ubiquinone: cytochrome c oxidoreductase apocytochrome b (mitochondrion)	Up	1.5944618	5.46311E–05
KAJ4215459.1	H^(+)^-transporting V0 sector ATPase subunit d	Up	1.50771061	8.00273E–05
KAJ4220368.1	H^(+)^-transporting V1 sector ATPase subunit F	Up	1.46590138	0.000231991
YP_005088120.1	NADH: ubiquinone oxidoreductase subunit 4 (mitochondrion)	Up	1.44701165	0.000686219
KAJ3463227.1	Hypothetical protein MRS44_008013	Up	1.40726655	0.000352133
KAJ4205864.1	Hypothetical protein NW759_014497	Up	1.33430929	2.67534E–05
KAJ3457463.1	Hypothetical protein MRS44_014604	Up	1.27888881	0.00182077
KAJ4229965.1	Hypothetical protein NW759_003329	Up	1.27721166	0.000161469
KAJ4219941.1	ndufs2, NADH ubiquinone oxidoreductase 49 kd subunit	Up	1.2524784	0.002220664
KAJ4224858.1	Hypothetical protein NW759_005567	Up	1.23747839	0.001197863
KAJ4218596.1	Mitochondrial acyl carrier protein	up	1.23605547	0.045710366
YP_005088119.1	NADH: ubiquinone oxidoreductase subunit 1 (mitochondrion)	Up	1.23506786	0.022195733
YP_005088100.1	NADH: ubiquinone oxidoreductase subunit 2 (mitochondrion)	Up	1.23336182	1.54084E–06
KAJ3465456.1	Hypothetical protein MRS44_006114	Up	1.22273977	0.000174469
YP_005088123.1	ATP synthase subunit 6 (mitochondrion)	Up	1.21093389	0.046734338
KAJ3468945.1	Hypothetical protein MRS44_003010	Down	0.82949938	0.014538644
KAJ4237375.1	ATP synthase F0 subcomplex subunit OSCP atp5	Down	0.81430181	0.002361697
KAJ4231083.1	Cytochrome c oxidase subunit 13, mitochondrial	Down	0.81350458	0.001045687
KAJ4201067.1	Cytochrome c oxidase subunit 7A	Down	0.80282109	0.015371909
KAJ4227226.1	atp18 subunit J of the mitochondrial F1F0 ATP synthase	Down	0.77282646	0.009143652
KAJ4215385.1	Cytochrome c oxidase assembly protein cox11, mitochondrial	Down	0.75984583	0.000482613
KAJ4220071.1	Inorganic pyrophosphatase	Down	0.71050994	3.03619E–05
KAJ4228229.1	atp2, beta subunit of the F1 sector of mitochondrial F1F0 ATP synthase	Down	0.70631362	0.000173542
KAJ4220045.1	ATP synthase F0 subcomplex subunit H atp14	Down	0.65402664	0.000259017
KAJ4220004.1	F1F0 ATP synthase subunit e, mitochondrial	Down	0.59724968	2.91446E–05
KAJ4235649.1	Cytochrome c oxidase subunit 6B	Down	0.5863684	1.18174E–07
KAJ4218594.1	ndufs8, ubiquinone oxidoreductase 23 kd subunit	Down	0.54946496	3.35858E–05
KAJ4229807.1	Delta subunit of the central stalk of mitochondrial F1F0 ATP synthase, atp16	Down	0.53590712	1.3247E–05
KAJ4225594.1	Hypothetical protein NW759_005287	Down	0.52417459	6.13201E–05
XP_046129462.1	Uncharacterized protein B0J15DRAFT_495975	Down	0.35837027	8.0906E–08
**Citrate cycle (TCA cycle)**				
KAJ3462447.1	Hypothetical protein MRS44_007233	Up	1.31003733	1.79225E–05
KAJ4228233.1	2-oxoglutarate dehydrogenase E1 component	Down	0.80221221	0.000132656
KAJ4205842.1	Dihydrolipoamide dehydrogenase precursor	Down	0.74787102	2.52016E–06
KAJ3467124.1	Hypothetical protein MRS44_004688	Down	0.72798332	2.02325E–05
KAJ3457638.1	Hypothetical protein MRS44_014779	Down	0.71028069	0.000343888
KAJ4219018.1	NADP-dependent isocitrate dehydrogenase	Down	0.69299592	0.000135752
KAJ4235919.1	Pyruvate carboxylase	Down	0.67526787	3.81328E–06
KAJ4232090.1	Hypothetical protein NW759_002482	Down	0.56780346	3.66E–07
KAJ4218387.1	ACONITATE hydratase mitochondrial	Down	0.54826728	3.56148E–07
KAJ4235797.1	Beta subunit of ATP citrate lyase	Down	0.50769447	7.06415E–06
KAJ4222063.1	Citrate (Si)-synthase	Down	0.44739185	2.61616E–06
KAJ4213686.1	Malate dehydrogenase, cytoplasmic	Down	0.39446474	3.27267E–06

### 3.7 Glycolysis/gluconeogenesis

**FIGURE 8:**
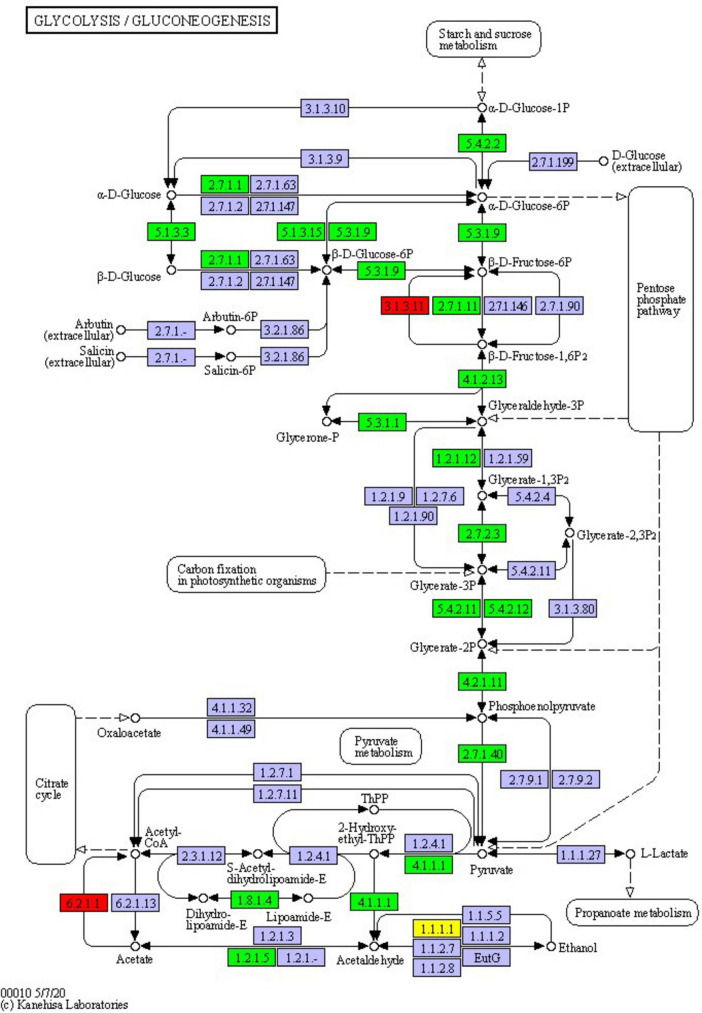
Changes in glycolysis/gluconeogenesis pathway of *F. solani* treated with PGA. Red and green proteins represent up-regulated and down-regulated protein expression levels.

The glycolysis/gluconeogenesis pathway is essential for the breakdown and synthesis of sugars, with glycolysis a primary pathway for glucose breakdown. This study’s results indicate that following PGA treatment, 24 DEPs were identified within the glycolysis and gluconeogenesis pathways of *F. solani*, comprising 3 upregulated and 21 downregulated proteins (Figure 8). Specifically, the expression levels of phosphoglucomutase, fructose-bisphosphate aldolase 1, and phosphoglycerate kinase were significantly down-regulated by factors of 0.39, 0.38, and 0.32, respectively, while the expression of fructose-1,6-bisphosphatase was significantly up-regulated by a factor of 1.01. These findings suggest that PGA treatment has a significant impact on the activity of the glycolysis/gluconeogenesis pathway.

### 3.8 Oxidative phosphorylation

**FIGURE 9:**
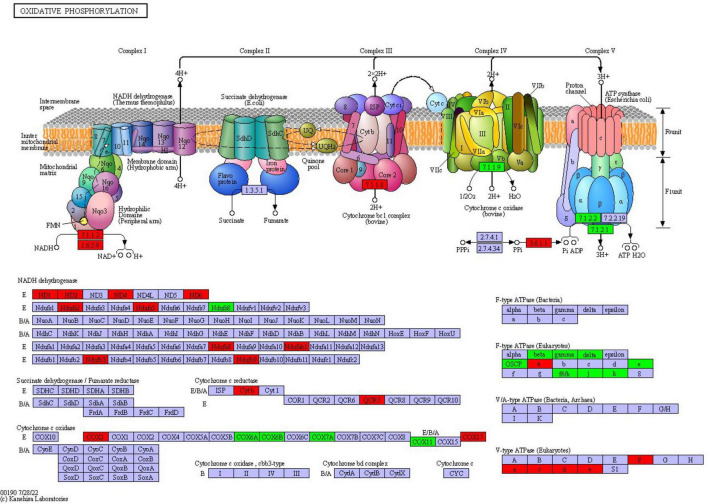
The changes of oxidative phosphorylation pathway of *F. solani* treated with PGA. Red and green proteins represent up-regulated and down-regulated protein expression levels.

Oxidative phosphorylation is the main way for fungi to obtain energy. Through electron transfer in the respiratory chain and the action of ATP synthase, the energy released during the oxidative decomposition of organic matter is converted into ATP which can be directly utilized by cells, providing a lot of energy for the growth, reproduction, metabolism and other life activities of fungi. The results of this study indicated that a total of 39 proteins were differentially expressed in the oxidative phosphorylation pathway of *F. solani*

following PGA treatment (Figure 9), with 24 proteins up-regulated and 15 proteins down-regulated. Notably, the expression of cytochrome c oxidase subunit 3 was significantly up-regulated by a factor of 3.06, while the ndufs8, ubiquinone oxidoreductase 23 kDa subunit, and delta subunit of the central stalk of mitochondrial F1F0 ATP synthase (atp16) were significantly down-regulated by factors of 0.55 and 0.53, respectively. These findings suggest that PGA treatment has a substantial impact on the oxidative phosphorylation pathway of *F. solani*.

### 3.9 TCA cycle

**FIGURE 10:**
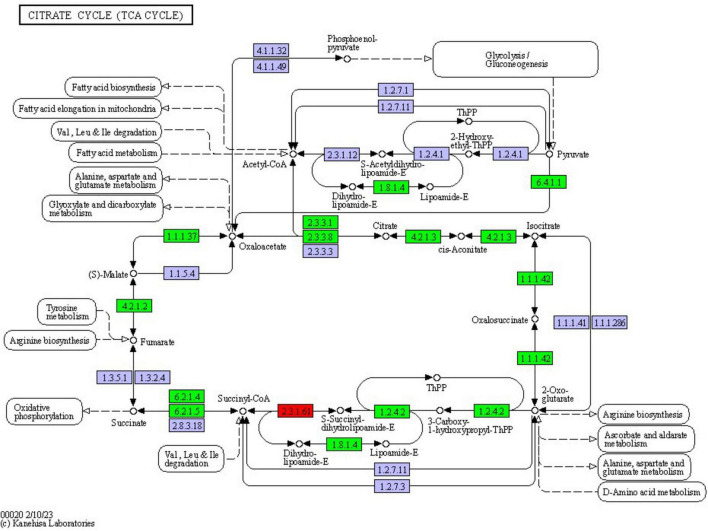
Changes in the tricarboxylic acid cycle pathway of *F. solani* treated with PGA. Red and green represent up-regulated and down-regulated protein expression levels.

Most of the differentially expressed proteins (DEPs) in the tricarboxylic acid (TCA) cycle pathway were found to be up-regulated. This study revealed that a total of 12 proteins exhibited differential expression in the TCA cycle pathway of *F. solani* following PGA treatment, with 11 proteins being up-regulated and 1 down-regulated (Figure 10). These findings indicate that PGA treatment enhances the expression of the beta subunit of ATP citrate lyase and citrate synthase within the TCA cycle pathway, thereby influencing the overall TCA cycle.

### 3.10 PRM and PCR validation of differential proteins

**FIGURE 11:**
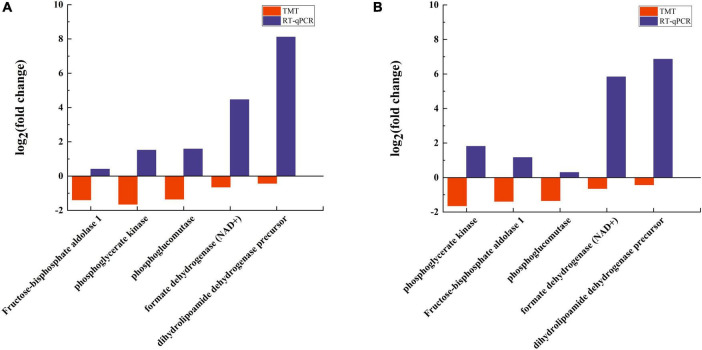
Validation of DEPs. **(A)** PRM validation of 5 differential proteins. **(B)** RT-qPCR verification of 5 genes.

According to the results of the pathway analysis related to energy metabolism, this study selected five DEPs for quantitative detection using PRM. The PRM quantitative results were compared with those obtained from TMT. The findings indicate that the quantitative results from PRM align with the overall trends observed in the TMT data; specifically, the down-regulated and up-regulated proteins identified through TMT also exhibited corresponding changes in PRM. Furthermore, the PRM results demonstrated more pronounced expression differences (Figure 11A), underscoring the stability and quantitative accuracy of both the PRM and TMT methodologies, thereby affirming the reliability of the TMT quantitative results.

To further validate the accuracy of the experimental outcomes, five proteins associated with energy metabolism were selected for analysis, and reverse transcription quantitative polymerase chain reaction (RT-qPCR) was employed to determine whether the transcription levels of the genes encoding these proteins were consistent with the proteomics findings. The results revealed that the expression trends of the five genes were similar to those observed in the TMT quantitative results, although the expression magnitudes differed (Figure 11B). This suggests that the RT-qPCR analysis corroborated the reliability of the proteomics analysis.

## 4 Discussions

We employ mitochondrial TMT proteomics technology to elucidate the effects of PGA on *F. solani* from a molecular biology perspective, specifically focusing on mitochondrial proteomics and its antifungal mechanisms. The results showed that after PGA treatment, the mycelium of *F. solani* showed swelling and curling, and the spore surface was significantly depressed. PGA has adverse effects on the surface structure of hyphae and spores of *F. solani*. Furthermore, DEPs were predominantly enriched in pathways related to energy metabolism, including carbohydrate metabolism, membrane components, intrinsic membrane components, and the endoplasmic reticulum, among other cellular components. These findings suggest that PGA inhibits the growth of *F. solani* by modulating the expression of key proteins involved in processes such as cell membrane synthesis, mitochondrial membrane biosynthesis, and energy metabolism.

Natural metabolite PGA exhibits a wide range of biological activities (Nenaah, 2010), yet its antifungal mechanism remains notably complex. Currently, the antifungal mechanism of PGA against pathogenic fungi has not been fully elucidated. Mitochondria, as critical organelles in eukaryotic cells, play a key role in regulating energy metabolism, biosynthesis, and cell death processes, including apoptosis and programmed necrosis (Fu et al., 2020; Guarente, 2008). They are integral to various physiological processes such as the tricarboxylic acid cycle, oxidative phosphorylation, pyruvate metabolism, fatty acid biosynthesis, and calcium homeostasis (Schrepfer and Scorrano, 2016). Tea tree essential oil disrupts the energy metabolism pathway of *Botrytis cinerea* by inhibiting glycolysis, impairing the tricarboxylic acid (TCA) cycle, and inducing mitochondrial dysfunction, which subsequently affects cellular life activities (Xu et al., 2017). A proteomics study investigating the inhibitory effects of perilla essential oil on *Aspergillus flavus* revealed that the DEPs induced by perilla essential oil were primarily enriched in amino acid biosynthesis and metabolism, antioxidant metabolism, fatty acid metabolism, lipid metabolism, and energy metabolism (Hu et al., 2022). Previous research has demonstrated that the antifungal mechanisms of plant-derived extracts are associated with their ability to disrupt the energy metabolism of pathogenic fungi.

Our study found that the majority of DEPs associated with the glycolysis pathway were down-regulated in *F. solani* treated with PGA, with the exception of fructose-1,6-bisphosphatase, which was up-regulated. The initial step in the glycolytic pathway involves the downregulation of hexokinase expression, followed by the downregulation of ATP-dependent 6-phosphofructokinase (pfka). Subsequently, triose phosphate isomerase (TPIA) is down-regulated, which catalyzes the conversion of dihydroxyacetone phosphate into 3-glyceraldehyde phosphate. Additionally, 3-glyceraldehyde phosphate is down-regulated, affecting the catalysis of 1,3-diphosphate to produce 3-glyceraldehyde 3-phosphate dehydrogenase (gapA) and phosphoglycerate kinase (PGK). All these proteins exhibited a downward trend, indirectly inhibiting the production of 2-phosphoglycerate. The expression of pyruvate kinase is also downregulated at a later stage, thereby limiting pyruvate synthesis. These findings align with previous studies (Lomora et al., 2017; Rashid et al., 2004; Ryu et al., 2006). Furthermore, two key enzymes, phosphoglucomutase (PGM) (Chen et al., 2021) and phosphoglycerate kinase (Chen et al., 2024), were also observed to have down-regulated expression. These results suggest that the antifungal effect of PGA is linked to the down-regulated expression of various key enzymes in the glycolysis pathway. Therefore, we infer that PGA treatment inhibits the normal progression of both glycolysis and gluconeogenesis pathways, thereby disrupting the energy metabolism of *F. solani.*

Oxidative phosphorylation is a crucial metabolic pathway. Through the phosphorylation of the electron transport chain, the energy released from the dehydrogenation of metabolites is utilized to catalyze the synthesis of ATP, thereby providing essential energy for the organism (Dimroth et al., 2000). In this study, cytochrome c oxidase (Yoshikawa and Shimada, 2015) was identified as a DEP, which is a key enzyme involved in ATP generation within the respiratory electron transport chain. Following PGA treatment, the expression of this enzyme exhibited a significant downward trend. Similar findings were observed in the ATPases detected, which catalyze the decomposition of ATP into ADP and phosphate ions (Kühlbrandt and Davies, 2016). Due to the down-regulation of ATPase and cytochrome c oxidase expression, even the up-regulation of NADH dehydrogenase expression is insufficient to meet ATP demands, thereby disrupting the carbohydrate, protein, and fat metabolism pathways associated with the tricarboxylic acid cycle. Consequently, it can be inferred that the antifungal activity of PGA against *F. solani* may be linked to the inhibition of ATP synthesis and normal energy metabolism. Additionally, oxidative phosphorylation is closely related to the TCA cycle. Our findings indicate that treatment of *F. solani* with PGA resulted in the down-regulation of pyruvate carboxylase (ALDH), which slowed the supply of oxaloacetate and disrupted the orderly progression of energy metabolism in *F. solani* due to the inhibition of the glycolytic pathway. Additionally, we observed that citric acid synthase is also down-regulated within the tricarboxylic acid cycle pathway, limiting the synthesis of citric acid from oxaloacetate and acetyl-CoA, thereby playing a crucial role in energy metabolism. Notably, the down-regulation of isocitrate lyase hampers the conversion of isocitrate into glyoxylic acid and succinic acid, further impeding the orderly progression of the tricarboxylic acid cycle. Glyoxylic acid, under the catalysis of malic acid synthase, reduced the content of malic acid, failing to provide a continuous and effective energy supply for PGA-treated *Aspergillus flavus*. Zhao et al. (2018) found that the expression of malate dehydrogenase was up-regulated after treatment with shenqinmycin in *Phoma* sp., and the activity of *Phoma* was inhibited by the increased activity of this enzyme. In conclusion, the inhibitory effect of PGA on *F. solani* may be linked to the disruption of its normal energy metabolism.

The trend in enzyme expression observed in RT-qPCR results diverges from that seen in TMT protein quantification. This discrepancy may account for the differences in transcriptional and translational levels of the target gene, with post-translational modification processes also influencing the expression of the final protein.

In summary, morphological and bioinformatics analyses indicate that PGA inhibits the growth of *F. solani* by modulating the expression of key enzymes involved in various energy metabolism pathways. We have elucidated the antifungal mechanism of PGA on *F. solani* in terms of energy metabolism from a proteomics perspective, thus providing a theoretical foundation for the application of PGA in plant disease control. Further investigations will be conducted to determine whether additional antifungal mechanisms exist.

## 5 Conclusion

A total of 2,412 differential proteins were screened by TMT proteomics, of which 1,083 were significantly up-regulated and 1,329 were significantly down-regulated. GO function analysis showed that the DEPs were mainly involved in integral component of membrane, intrinsic component of membrane, pyridine-containing compound metabolic process, carbon-oxygen lyase activity, integral component of membrane, intrinsic component of membrane, and endoplasmic reticulum. The results of KEGG enrichment analysis showed. DEPs were mainly enriched in pentose and glucuronate interconversions, propanoate metabolism, various types of N-glycan biosynthesis, pentose phosphate pathway, carbon fixation in photosynthetic organisms and other important pathways. The quantitative results of PRM were consistent with the overall trend of TMT quantitative results, and the results of PRM verification were more significant in expression, indicating that TMT quantitative results were reliable. The results of RT-qPCR showed that the expression trend of 5 genes was consistent with the results of TMT quantification. Therefore, the results of RT-qPCR analysis supported the reliability of proteomics analysis results. PGA inhibited the growth of *F. solani* mitochondria by affecting its energy metabolism.

## Data Availability

The original contributions presented in the study are publicly available. This data can be found here: ProteomeXchange Consortium database (https://proteomecentral.proteomexchange.org/cgi/GetDataset?ID=PXD058927) under the accession number, PXD058927.

## Footnotes

1
https://www.iprox.cn
